# Li_2_Ca_1.5_Nb_3_O_10_ from X-ray powder data

**DOI:** 10.1107/S160053681100688X

**Published:** 2011-03-12

**Authors:** Bai-Chuan Zhu, Kai-Bin Tang

**Affiliations:** a Department of Chemistry, University of Science and Technology of China, Hefei, Anhui 230026, People’s Republic of China; b Department of Nanomaterial and Nanochemistry, Hefei National Laboratory for Physical Sciences at Microscale, University of Science and Technology of China, Hefei, Anhui 230026, People’s Republic of China

## Abstract

Lithium calcium niobium oxide (2/1.5/3/10), Li_2_Ca_1.5_Nb_3_O_10_, has been synthesized by conventional solid-state reaction. Its structure consists of triple-layer perovskite slabs of corner-sharing NbO_6_ octa­hedra inter­leaved with lithium ions; Ca cations partially occupy the perovskite *A* sites at 75% occupancy probability. All eight atoms in the asymmetric unit are on special positions: one Nb atom has site symmetry 4/*mmm*; the second Nb, both K, the Sr and two O atoms have site symmetry 4*mm*; the remaining two O atoms have site symmetries 2*mm*. and *mmm*., respectively.

## Related literature

For background to Ruddlesden–Popper layered perovskites, see: Schaak & Mallouk (2002 ▶). Structures of related crystal *A-*site deficient three-layer Ruddlesden–Popper phases have been reported for K_2_Sr_1.5_Ta_3_O_10_ (Le Berre *et al.*, 2002 ▶), Li_4_Sr_3_Nb_6_O_20_ (Bhuvanesh *et al.*, 1999*a* ▶), Li_2_La_1.78_Nb_0.66_Ti_2.34_O_10_ (Bhuvanesh *et al.*, 1999*b* ▶) and Li_2_CaTa_2_O_7_ (Liang *et al.*, 2008 ▶). For crystallographic background, see: Howard (1982 ▶); Thompson *et al.* (1987 ▶).

## Experimental

### 

#### Crystal data

Li_2_Ca_1.5_Nb_3_O_10_
*M* *_r_* = 512.71
Tetragonal, 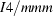

*a* = 3.87880 (6) Å
*c* = 26.2669 (4) Å
*V* = 395.19 (1) Å^3^
*Z* = 2
Cu *K*α radiation, λ = 1.54060, 1.54443 Å
*T* = 298 K
flat sheet, 20 × 20 mm

#### Data collection

PANalytical X’pert PRO diffractometer
Specimen mounting: packed powder pellet
Data collection mode: reflection
Scan method: continuous
2θ_min_ = 10.004°, 2θ_max_ = 129.939°, 2θ_step_ = 0.017°

#### Refinement

*R*_p_ = 0.050
*R*_wp_ = 0.076
*R*_exp_ = 0.009
*R*(*F* ^2^) = 0.068
χ^2^ = 0.706
7056 data points
51 parameters

### 

Data collection: *X’pert Data Collector* (PANalytical, 2003 ▶); cell refinement: *GSAS* (Larson & Von Dreele, 2004 ▶) and *EXPGUI* (Toby, 2001 ▶); data reduction: *X’pert Highscore* (PANalytical, 2003 ▶); method used to solve structure: coordinates taken from an isotypic compound (Bhuvanesh *et al.*, 1999*a*; Liang *et al.*, 2008 ▶); program(s) used to refine structure: *GSAS* and *EXPGUI*; molecular graphics: *DIAMOND* (Brandenburg, 1999 ▶); software used to prepare material for publication: *publCIF* (Westrip, 2010 ▶).

## Supplementary Material

Crystal structure: contains datablocks global, I. DOI: 10.1107/S160053681100688X/vn2001sup1.cif

Rietveld powder data: contains datablocks I. DOI: 10.1107/S160053681100688X/vn2001Isup2.rtv

Additional supplementary materials:  crystallographic information; 3D view; checkCIF report

## Figures and Tables

**Table 1:** Selected bond lengths (Å)

Nb1—O1^i^	1.9394 (1)
Nb1—O4	2.027 (11)
Nb2—O2	1.689 (8)
Nb2—O3^i^	1.9704 (11)
Nb2—O4	2.029 (11)
Ca1—O1^ii^	2.805 (4)
Ca1—O3^ii^	2.567 (4)
Ca1—O4^iii^	2.7427 (1)
Li1—O2	1.599 (4)

Symmetry codes: (i) 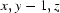; (ii) 
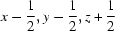
; (iii) 
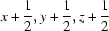
.

**Fig. 1.**
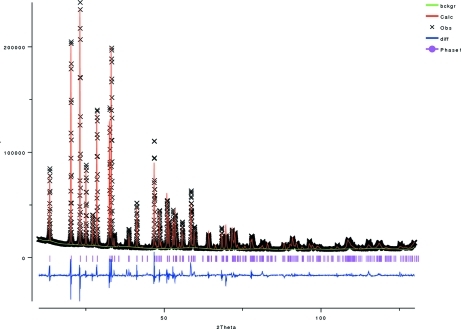
Experimental and calculated X-ray diffraction pattern of Li2Ca1.5Nb3O10. The difference profile is given at the bottom. The Bragg positions are indicated by the vertical markers below the observed pattern.

**Fig. 2.**
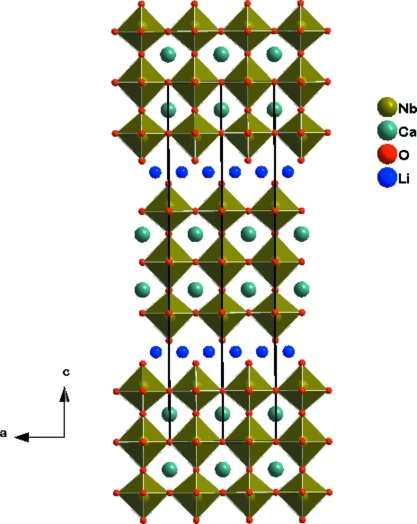
The crystal structure of Li2Ca1.5Nb3O10 in a projection along [010].

## supplementary crystallographic information

### Comment

Layered perovskites that belong to the Ruddlesden-Popper family have a general formula *A'*_2_[*A*_n-1_*B*_n_O_3n+1_] (Schaak *et al.*, 2002), where *B* is a small transition metal cation, *A* is a larger *s*-, *d*-, or *f*-block cation and *A'* is always an alkali cation. The Ruddlesden-Popper phases which are intergrowths of the perovskite and rocksalt structures posses a wide variety of interesting properties including superconductivity, colossal magnetoresistance, ferroelectricity, and catalytic activity. Related crystal structures of *A* sites deficiency three-layer Ruddlesden-Popper phases have been reported for K_2_Sr_1.5_Ta_3_O_10_(Le Berre *et al.*, 2002), Li_2_La_1.78_Nb_0.66_Ti_2.34_O_10_ ( Bhuvanesh *et al.*, 1999*b*), and Li_4_Sr_3_Nb_6_O_20_ ( Bhuvanesh *et al.*, 1999*a*).

Fig. 1 shows the observed, calculated and difference plots of the Rietveld refinement. We applied the March-Dollase formalism for a correction of the 00*l* preferential orientation which is frequently observed in Rietveld refinement of layered perovskites.

The structure of the compound is illustrated in Fig. 2. It is formed from two differently stacked NbO_6_ octahedra thick slabs cut along the c direction. Two successive layers are shifted by (*a*+*b*)/2 with Ca cations partially occupying the 12-coordinated sites. The Li cations occupy the interlayer spacing at Wyckoff site 8*f* and not the 4*e* site since the distance between two adjacent layers is short. Ca cations partially occupy the perovskite *A* sites at 75% occupancy probability. The Nb cations are coordinated by six oxygen atoms to form NbO_6_ octahedra with Nb—O distances ranging from 1.689 (8) to 2.029 (11) Å. The octahedra forming the outer layer of the slabs are characterized by off-centering of the Nb atoms, leading to four equal equatorial Nb—O distances within the perovskite layers [1.9704 (11) Å], a short Nb—O bond toward the interlayer spacing [1.689 (8) Å], and a long opposite Nb—O bond [2.029 (11) Å]. The octahedra forming the inner layer are less distorted with four equal equatorial Nb—O distances[1.9394 (1) Å] and other two equal Nb—O distances [2.027 (11) Å] parallel to the *c* axis. These type of distorsions are well known in triple-layer perovskites.

### Experimental

The sample was prepared by conventional solid-state reaction. Stoichiometric amounts of Li_2_CO_3_,CaCO_3_ and Nb_2_O_5_ were mixed, ground, and calcined at 1423 K for 6 h with one intermediate grid. An excess amount of Li_2_CO_3_(20 mol%) was added to compensate for the loss due to the volatilization of alkali metal carbonate.

### Refinement

The crystal structures of Li_4_Sr_3_Nb_6_O_20_ (Bhuvanesh *et al.*, 1999*a*) and Li_2_CaTa_2_O_7_ (Liang *et al.*, 2008) were used as a starting model for the Rietveld refinement. The X-ray powder diffraction patterns of Li_2_Ca_1.5_Nb_3_O_10_ were indexed in a body-centered tetragonal space group *I*4/*mmm*. Structure refinement was carried out by the Rietveld method using the *GSAS* profile refinement program (Larson & Von Dreele, 2004). The site occupancy factors of Ca and Li were set at 0.75 and 0.50, respectively in view of the close ressemblance of the cell parameters with those of the related structures and they were not further refined. The corresponding isotropic atomic displacement parameters of all oxygen atoms and niobium atoms were constrained to be equal, respectively. The March-Dollase option in the EXPGUI program (Toby, 2001) was applied to correct 00*l* preferential orientation.

### Crystal data

**Table:**

Li_2_Ca_1.5_Nb_3_O_10_	*Z* = 2
*M**_r_* = 512.71	*D*_x_ = 4.309 Mg m^−^^3^
Tetragonal, *I*4/*m**m**m*	Cu *K*α radiation, λ = 1.540600, 1.544430 Å
Hall symbol: -I 4 2	*T* = 298 K
*a* = 3.87880 (6) Å	white
*c* = 26.2669 (4) Å	flat sheet, 20 × 20 mm
*V* = 395.19 (1) Å^3^	Specimen preparation: Prepared at 1423 K

### Data collection

**Table:**

PANalytical X'pert PRO diffractometer	Data collection mode: reflection
Radiation source: sealed tube	Scan method: continuous
graphite	2θ_min_ = 10.004°, 2θ_max_ = 129.939°, 2θ_step_ = 0.017°
Specimen mounting: packed powder pellet	

### Refinement

**Table:**

Refinement on *F*^2^	Profile function: CW Profile function number 2 with 18 terms Profile coefficients for Simpson's rule integration of pseudovoigt function C.J. Howard (1982). J. Appl. Cryst.,15,615-620. P. Thompson, D.E. Cox & J.B. Hastings (1987). J. Appl. Cryst.,20,79-83. #1(GU) = 149.621 #2(GV) = -120.364 #3(GW) = 31.573 #4(LX) = 1.000 #5(LY) = 17.840 #6(trns) = 0.000 #7(asym) = 0.0000 #8(shft) = 0.0000 #9(GP) = 0.000 #10(stec)= 0.00 #11(ptec)= 0.00 #12(sfec)= 0.00 #13(L11) = 0.000 #14(L22) = 0.000 #15(L33) = 0.000 #16(L12) = 0.000 #17(L13) = 0.000 #18(L23) = 0.000 Peak tails are ignored where the intensity is below 0.0010 times the peak Aniso. broadening axis 0.0 0.0 1.0
Least-squares matrix: full	51 parameters
*R*_p_ = 0.050	0 restraints
*R*_wp_ = 0.076	4 constraints
*R*_exp_ = 0.009	*w* = 1/[σ^2^(*F*_o_^2^) + (0.0677*P*)^2^] where *P* = (*F*_o_^2^ + 2*F*_c_^2^)/3
*R*(*F*^2^) = 0.06796	(Δ/σ)_max_ = 0.01
χ^2^ = 0.706	Background function: GSAS Background function number 1 with 36 terms. Shifted Chebyshev function of 1st kind 1: 10229.8 2: -3178.07 3: 2423.18 4: -808.112 5: 540.944 6: -198.924 7: 271.065 8: 94.4177 9: 234.644 10: 188.507 11: 146.243 12: 265.504 13: -11.6147 14: 51.8836 15: 137.742 16: 26.3316 17: -53.6065 18: 3.80136 19: 279.859 20: -56.8162 21: -60.3405 22: 50.5886 23: 41.8504 24: 9.38150 25: -48.8258 26: -20.5686 27: -49.8098 28: 74.7145 29: -37.5745 30: 90.5252 31: -21.2918 32: -56.1545 33: 0.932266 34: -17.8446 35: -27.9120 36: -2.66006
7056 data points	Preferred orientation correction: March-Dollase AXIS 1 Ratio= 0.89341 h= 0.000 k= 0.000 l= 1.000 Prefered orientation correction range: Min= 0.84444, Max= 1.40236
Excluded region(s): none	

### Fractional atomic coordinates and isotropic or equivalent isotropic displacement parameters (Å^2^)

**Table:**

	*x*	*y*	*z*	*U*_iso_*/*U*_eq_	Occ. (<1)
Nb1	0.0	0.0	0.0	0.0112 (3)*	
Nb2	0.0	0.0	0.15442 (5)	0.0112 (3)*	
Ca1	0.0	0.0	0.5771 (2)	0.0157 (3)*	0.75
O1	0.0	0.5	0.0	0.0132 (3)*	
O2	0.0	0.0	0.2187 (3)	0.0132 (3)*	
O3	0.0	0.5	0.1412 (2)	0.0132 (3)*	
O4	0.0	0.0	0.0772 (4)	0.0132 (3)*	
Li1	0.25	0.25	0.25	0.0182 (3)*	0.5

### Geometric parameters (Å, °)

**Table:**

Nb1—O1^i^	1.939400 (30)	Ca1—O1^ii^	2.805 (4)
Nb1—O4	2.027 (11)	Ca1—O3^ii^	2.567 (4)
Nb2—O2	1.689 (8)	Ca1—O4^iii^	2.74272 (4)
Nb2—O3^i^	1.9704 (11)	Li1—O2	1.599 (4)
Nb2—O4	2.029 (11)		
			
O1^i^—Nb1—O1	180.0	O1^ii^—Ca1—O3^vi^	118.25 (6)
O1^i^—Nb1—O1^iv^	90.0	O1^ii^—Ca1—O4^ii^	60.74 (21)
O1^i^—Nb1—O4	90.0	O1^ii^—Ca1—O4^v^	119.28 (27)
O2—Nb2—O3^i^	100.17 (18)	O1^v^—Ca1—O3^v^	87.19 (9)
O2—Nb2—O4	180.0	O3^ii^—Ca1—O3^v^	98.14 (22)
O3^i^—Nb2—O3	159.7 (4)	O3^ii^—Ca1—O3^vi^	64.58 (12)
O3^i^—Nb2—O3^iv^	88.21 (6)	O3^ii^—Ca1—O4^vii^	57.70 (19)
O3^i^—Nb2—O4	79.83 (18)	O3^ii^—Ca1—O4^v^	122.28 (26)
O1^ii^—Ca1—O1^v^	87.49 (16)	O4^ii^—Ca1—O4^vii^	90.00000 (20)
O1^ii^—Ca1—O1^vi^	58.54 (9)	O4^ii^—Ca1—O4^iii^	180.00000 (30)
O1^ii^—Ca1—O3^v^	174.68 (17)	O2—Li1—O2^viii^	180.0

Symmetry codes: (i) *x*, *y*−1, *z*;  (ii) *x*−1/2, *y*−1/2, *z*+1/2; (iii) *x*+1/2, *y*+1/2, *z*+1/2; (iv) −*y*, *x*, *z*; (v) *x*+1/2, *y*−1/2, *z*+1/2; (vi) −*y*+1/2, *x*−1/2, *z*+1/2; (vii) *x*−1/2, *y*+1/2, *z*+1/2; (viii) −*x*−1/2, −*y*−1/2, −*z*−1/2.
